# Recent progress in understanding the role of ecdysteroids in adult insects: Germline development and circadian clock in the fruit fly *Drosophila melanogaster*

**DOI:** 10.1186/s40851-015-0031-2

**Published:** 2015-11-02

**Authors:** Outa Uryu, Tomotsune Ameku, Ryusuke Niwa

**Affiliations:** Graduate School of Life and Environmental Sciences, University of Tsukuba, Tennoudai 1-1-1, Tsukuba, Ibaraki 305-8572 Japan; Faculty of Life and Environmental Sciences, University of Tsukuba, Tennoudai 1-1-1, Tsukuba, Ibaraki 305-8572 Japan; PRESTO, Japan Science and Technology Agency, Honcho 4-1-8, Kawaguchi, Saitama 332-0012 Japan

**Keywords:** Ecdysone, Steroid hormone, Insect, Germline stem cell, Oogenesis, Circadian clock

## Abstract

Steroid hormones are one of the major bioactive molecules responsible for the coordinated regulation of biological processes in multicellular organisms. In insects, the principal steroid hormones are ecdysteroids, including 20-hydroxyecdysone. A great deal of research has investigated the roles played by ecdysteroids during insect development, especially the regulatory role in inducing molting and metamorphosis. However, little attention has been paid to the roles of these hormones in post-developmental processes, despite their undisputed presence in the adult insect body. Recently, molecular genetics of the fruit fly *Drosophila melanogaster* has revealed that ecdysteroid biosynthesis and signaling are indeed active in adult insects, and involved in diverse processes, including oogenesis, stress resistance, longevity, and neuronal activity. In this review, we focus on very recent progress in the understanding of two adult biological events that require ecdysteroid biosynthesis and/or signaling in *Drosophila* at the molecular level: germline development and the circadian clock.

## Introduction

Steroid hormones play indispensable roles in modulating a broad range of biological processes in nearly all multicellular organisms [1–3]. Steroid hormones are biosynthesized from sterols, such as cholesterol, by members of specific steroidogenic enzymes in specialized steroidogenic tissues [4]. Once produced, steroid hormones are circulated in hemolymph and are easily transported to target cells to act as ligands for the nuclear receptor family of transcription factors [5]. The steroid hormone-nuclear receptor complexes affect gene expression in target cells, triggering a hormone-dependent response.

In insects, the major steroid hormones are ecdysteroids, also known as molting hormones. Ecdysteroids, especially the most biologically active form 20-hydroxyecdysone (20E), play essential roles in coordinating developmental transitions, such as larval molting and metamorphosis [2, 6]. 20E activates a heterodimeric nuclear hormone receptor complex of proteins encoded by the *Ecdysone receptor* (*EcR*) and *ultraspiracle* (*usp*) genes [7–10]. This heterodimer regulates the expression of ecdysone-responsive genes by binding to specific promoter sequences called ecdysone response elements. In contrast to the long history of studies of EcR/USP and its downstream gene cascades, identification and characterization of ecdysteroidogenic enzymes have only been achieved within the last 15 years. So far, there are at least 10 essential ecdysteroidogenic enzymes that are expressed in ecdysteroidogenic tissues/organs, such as the larval prothoracic gland (PG), during embryonic and larval development [2].

The timing of molting and metamorphosis are mainly determined by dynamic temporal fluctuations of hemolymph ecdysteroid pulses and the subsequent activation of the ecdysteroid-dependent gene cascade [11]. Previous studies have also demonstrated that many genetic mutants of *EcR*, *usp*, ecdysteroid-inducible genes and ecdysteroidogenic enzyme genes exhibit clear defects of molting and/or metamorphosis [2, 12]. Therefore, a large body of literature has described the roles of ecdysteroids to trigger such drastic developmental changes. By contrast, whereas a low but significant amount of ecdysteroids are undoubtedly present in adult stages, temporal changes of the hemolymph titer are ill-defined [13]. Furthermore, after the completion of development, the adult insects no longer display visible changes of either morphology or physiology. Perhaps for these reasons little attention had been paid to the functions of ecdysteroids in adult insects.

In the past decade, however, molecular genetic studies using *Drosophila* have revealed some important aspects of ecdysteroids in adult physiology [14]. In this review, we specifically focus on very recent progress in understanding two adult biological events that require ecdysteroid biosynthesis and/or signaling in *Drosophila* at the molecular level: germline development and the circadian clock.

## Review

### Oocyte maturation and ecdysteroids: Stage-8 checkpoint and lipid accumulation

The first evidence showing the role of ecdysteroids in adult insects was reported by studies using the ovaries of adult mosquitoes in the 1970s [15, 16]. These studies demonstrated that vitellogenin synthesis in the fat body of mosquitoes is regulated by ovarian ecdysteroids [16]. After this discovery, genetic studies of ecdysteroids and oogenesis have mainly been conducted using the convenient genetic model, the fruit fly *D. melanogaster*.

In *Drosophila*, ecdysteroids are also detected in the adult ovary [13, 17–19]. Genetic studies using mutants of genes required for ecdysteroid biosynthesis have proved that ovarian ecdysteroids are biosynthesized in the ovary itself. For example, adult females with a temperature-sensitive allele of *ecdysoneless* have a low ecdysteroid titer in the ovary [19, 20]. More recently, identification and characterization of a number of ecdysteroidogenic enzyme genes have enabled researchers to show that these genes are expressed in nurse cells and/or follicle cells of the adult ovary [19, 21–30]. Genetic studies have also confirmed that at least two of the ecdysteroidogenic genes, *spook* and *phantom*, are required for proper development of the ovary [27, 31]. In addition, some ecdysone response genes also play essential roles in oogenesis [32].

**Fig. 1:**
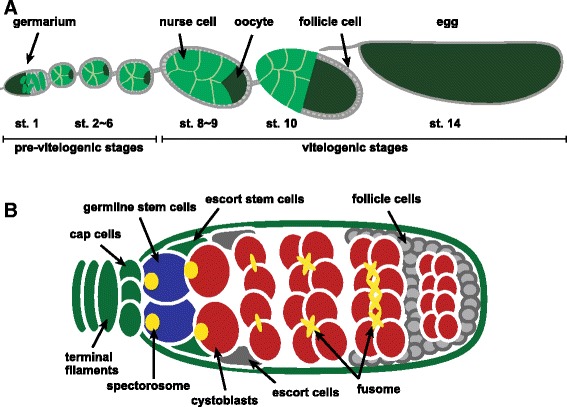
Schematic representation of ovariole and germarium in *Drosophila melanogaster*. **a** The *Drosophila* ovary is composed of 15–20 ovarioles. The continuous developing egg chamber is divided into 14 stages. Each egg chamber is composed of an oocyte, nurse cells and somatic follicle cells. Vitellogenesis occurs after stage 8 egg chamber. **b** The germarium resides in the tip of the ovariole. Germline stem cells (blue) are maintained by somatic niche cells comprising the terminal filament, cap cells, and escort stem cells (green). Germline stem cells produce another stem cell by self-renewal and also divide asymmetrically to produce daughter cells called cystoblasts (red). The cystoblast divides four times with incomplete cytokinesis to form 15 nurse cells and one oocyte in each egg chamber, which are enveloped by follicle cells (gray). Illustration in the egg chamber shows proliferation and differentiation of cystoblasts from the 2-(left) to 16-cell stage. GSCs and cystoblasts can be identified by the morphology of the spectrosome, a germline-specific membranous organelle (yellow). Developing cystocytes contain the fusome, a derivative of the spectrosome that shows more branched morphology (yellow)

**Fig. 2:**
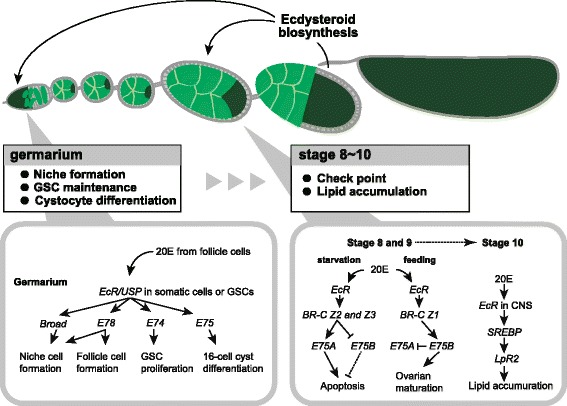
Different roles of ecdysteroids in regulating progression of oogenesis. Ecdysteroid biosynthesized in the stage 10 follicle cells regulates many aspects of oogenesis to function in the early and mid-stage of the egg chamber. Stage 8 checkpoint is determined by nutritional status and regulated by *E75A* and *E75B*. Starvation leads to apoptosis of the egg chamber via *E75A*, whose expression is negatively regulated by *E75B* under feeding conditions Ecdysteroid signaling in the CNS mediates lipid accumulation at stage 10 egg chamber via *SREBP* and *LpR2*. Ecdysteroids also function in early oogenesis at the germarium such as niche cell formation, follicle cell formation, GSC maintenance and cyst cell differentiation. *EcR/USP* are expressed in the somatic niche cells or GSCs to control different ecdysone responsive genes. While *E74* controls GSC proliferation, *E75* affects 16-cell cyst differentiation. *Broad* and *E78* regulate niche cell formation during ovarian development in late larval stages

While versatile roles of ecdysteroid signaling in the development of the ovary of *Drosophila* have been proposed [16], one important role is to act as a developmental checkpoint during mid-oogenesis to ensure proper egg production. The ovary of *Drosophila* is composed of 15–20 ovarioles that have continuously developing egg chambers [33] (Figure 1a). Each egg chamber can be divided into 14 stages based on morphological criteria. Stage 14 is the mature egg, and stage 1 is budding of the egg chamber in the anterior of ovarioles, called the germarium (Fig. 1a). During *Drosophila* oogenesis, there is a critical developmental checkpoint around stage 8 [34]. In this stage, a developmental decision is made in each egg chamber to determine whether it will develop or die. While a low concentration of ecdysteroids is essential for normal oogenesis, a high concentration of ecdysteroids caused by nutritional shortage induces apoptosis in the nurse cells of stage 8 and 9 egg chambers [34, 35]. In this checkpoint, *ecdysone-induced protein 75* (*E75*) isoforms are involved in inducing or suppressing apoptosis. While overexpression of *E75A* in the egg chamber induces apoptosis of the nurse cells at stages 8 and 9 in fed flies, overexpression of *E75B* suppresses it at stages 8 and 9 in starved flies, suggesting that *E75A* and *E75B* have the opposite effect on apoptosis: *E75A* induces apoptosis and *E75B* inhibits apoptosis [36]. In addition, expression of *E75* isoforms is regulated by the *BR-C* isoform. BR-C *Z2* and *Z3* are not expressed in the egg chambers at stages 8 and 9 under feeding conditions, but are expressed in the follicle cells under apoptotic conditions. Overexpression of *BR-C Z2* or *Z3* induces *E75A* expression and suppresses *E75B* expression in the egg chambers at stages 8 and 9 [36]. This suggests that *BR-C* isoforms respond to the nutritional signals and regulate the expression of *E75A* and *E75B* expression to control apoptosis in the stage 8 egg chamber (Fig. 2).

Notably, the ecdysteroid-dependent mid-oogenesis checkpoint is also influenced by organismal metabolism and external nutrient conditions, as illustrated by a recent study [37]. During oogenesis, lipids are maternally supplied to oocytes and the lipid storage is crucial for the early stages of embryogenesis in many animals [38, 39]. In *Drosophila*, lipids accumulate in the stage 10 oocyte via a low-density lipoprotein (LDL) receptor. A recent study has demonstrated that ecdysteroid signaling is required for lipid accumulation, and feeding behavior is required for proper nutrition uptake (Fig. 2) [37]. *EcR* mutant females have a defect in lipid accumulation and exhibit reduced levels of the LDL receptor *LpR2*. The expression of *LpR2* is regulated by *Sterol regulatory element-binding proteins* (*SREBP)*, the important lipogenic transcription factor in response to ecdysteroid signaling and dietary nutrients. In addition, adult-specific dominant-negative *EcR* expression in the central nervous system (CNS) causes decreased levels in feeding behavior and nutrient uptake in females. As oral administration of 20E induces nutrient storage [37], it is possible that ecdysteroid signaling in the CNS may promote nutrient accumulation required for the proper level of egg laying in females. However, it is unclear how follicle cells perceive nutrient information regarding starvation or feeding of individuals to control ecdysteroid levels.

### Germline stem cells and ecdysteroids

In addition to the previously reported ecdysteroid-dependent regulation of oogenesis, such as in mid-oogenesis as described above, as well as oocyte maturation and oviposition [32, 40], recent studies have revealed that ecdysteroids also control very early steps of oogenesis, namely niche formation, germline stem cell (GSC) behavior, and cyst cell differentiation.

In the germarium in adult *Drosophila* females, 1–3 GSCs give rise to mature eggs (Fig. 1b). GSCs reside in a specialized microenvironment called the niche that maintains stem cell function by sending local niche signals into GSCs and controls symmetric or asymmetric GSC division [41, 42]. GSCs can divide symmetrically to produce daughter stem cells, or asymmetrically to produce daughter cells called cystoblasts that differentiate into nurse cells and oocytes. The cystoblast undergoes four mitotic divisions with incomplete cytokinesis to form 15 nurse cells and one oocyte in each egg chamber that is surrounded by somatic follicle cells.

The ovary of *Drosophila* has long been recognized as one of the most powerful tools for investigating GSCs and niches [43]*.* GSCs receive a somatic signal from niches consisting of the terminal filament and cap cells, which maintain GSC function (Fig. 1b). In the larval ovary, both primordial germ cells (PGC, the precursors of GSCs) and gonadal somatic cells (the precursors of niche cells) proliferate and develop to form 16–20 GSC units of the adult ovary [44]. Ecdysteroid signaling controls formation of niche and stem cell precursors in the larval ovarian development. Although knocking down of *EcR* or *Usp* function in the somatic ovary at the early third instar does not change developmental timing, precocious differentiation of both niches and PGCs occurs in gonads of *EcR* or *usp* RNAi animals at the early third instar [45]. However, overexpression of the dominant negative form of *EcR* at the mid-third instar causes reduced size in the ovary and niche [45]. These results suggest that the ecdysone receptor represses precocious differentiation of both niches and PGCs at the early third instar, and is required for niche formation and gonadal development at the mid-third instar (Fig. 2). In addition, this mechanism involves the early ecdysone response gene, *Broad-Z1*. Ecdysteroid signaling non-cell-autonomously activates *Broad* expression in the somatic ovary through *EcR*/*Usp* to form niche and differentiating PGCs at the mid-third instar and later, but not the early third instar [45]. In addition, loss of ecdysone-induced transcription factor, *E78* results in decreased cap cell numbers and fewer germline stem cells [46], suggesting that ecdysteroid signaling controls niche assembly to maintain the proper number of GSCs via *E78* and *Broad* (Fig. 2).

GSCs are maintained by local niche signals and are also affected by systemic ecdysteroid signaling. *EcR* mutant females show a reduced number of GSCs independent of insulin signaling, suggesting that ecdysteroid signaling directly regulates adult GSC proliferation and self-renewal [47]. This regulation is mediated by *E74*, a transcription factor known as an early responsive gene of ecdysteroids, while other transcription factors, *E75* and *BR-C*, are not required for proper GSC proliferation (Fig. 2) [47]. Moreover, ecdysteroid signaling controls GSC proliferation by interacting with chromatin remodeling factors such as ISWI (an intrinsic epigenetic factor required for GSC fate and activity) and Nurf301 (the largest subunit of the ISWI-containing NURF chromatin remodeling complex), suggesting that there is an essential link between ecdysteroid signaling and the intrinsic chromatin remodeling machinery as a potential mechanism for promoting the general transcriptional program [47].

GSCs undergo four rounds of synchronous division to produce 2, 4, 8, and eventually 16 interconnected developing cysts (called cystocytes), the precursors of ovarian follicles. Somatic follicle cells envelop each cystocyte to form a follicle through 14 developmental stages and support proper differentiation. Ecdysteroid signaling is also required for cyst differentiation. Overexpression of the dominant-negative form of *EcR* in somatic escort cells that envelop the GSC progeny disrupts early germ cell differentiation [48]. In addition, mutants for ecdysteroid signaling pathway components in escort cells show increased levels of the cell adhesion molecules β-Catenin/Armadillo, *D*E-Cadherin and a cytoskeleton component Adducin [48]. These data suggest that ecdysteroid signaling in somatic escort cells plays an important role in controlling germ cell differentiation via regulation of cell adhesion complexes required for the establishment of physiological germline–soma interaction [48, 49]. Moreover, knocking down the components of *EcR* or *E75* in escort cells causes a reduced number of 16-cell cysts, but not 2-, 4- and 8-cell cysts and disrupted follicle cell formation, suggesting that ecdysteroid signaling has a specific role in controlling entry into meiosis of 16-cell cysts (Fig. 2) [50]. In addition, mutants for *E78* show a significant decrease in ovarian follicle cell numbers, suggesting that ecdysteroid signaling is also required for follicle cell survival (Fig. 2) [46].

In addition to the ovary, ecdysteroids are also detected in the testis [13, 17, 18]. The role of ecdysteroids in stem cell maintenance in the testis has been reported recently. The *Drosophila* testis stem cell niche consists of a cluster of non-mitotic somatic cells called the hub, which produces signals that maintain surrounding GSCs as well as cyst stem cells (CySCs). CySCs produce cyst cells that are required for differentiation to sperm from GSC daughters. In this system, ecdysteroid signaling pathway components are essential for the maintenance and survival of both GSCs and CySCs [51]. Moreover, as well as the ovarian GSC system, *EcR* genetically interacts with Nurf301 to maintain these stem cells in the testis niche. These results imply that ecdysteroid signaling is required for stem cell maintenance beyond sexes at least in *Drosophila* [51].

### Ecdysteroid signaling factors in the molecular machinery of the circadian clock

Besides the germline, which is the most classical site of action of ecdysteroids in adult insects, ecdysteroids also influence many other adult organs and tissues. Recent studies have shown that ecdysteroids are involved in adult neuronal function, including the control of learning, memory, and behavior [52–55]. Particularly, very recent studies have unraveled the ecdysteroid-dependent regulation of circadian clocks in insects, especially *Drosophila*.

Circadian clocks coordinate rhythmic behaviors and help living organisms adapt to the daily cycling of environmental conditions [56]. Circadian clocks provide the obvious advantage of anticipatory preparation for predictably recurrent conditions, which cannot be achieved by direct responses to conditions that have already commenced. The molecular machinery of the circadian clock has been extensively studied in *Drosophila*, where the circadian master clock comprises about 150 neurons located in the central brain [57]. The oscillation of the clock is thought to be generated by a molecular mechanism that is composed of transcriptional-translational autoregulatory feedback loops of the clock genes, such as *period* (*per*), *timeless* (*tim*) *Clock* (*Clk*), and *cycle* (*cyc*) [58, 59]. The CLK-CYC heterodimer directly activates transcription of *per*, *tim*, *vrille* (*vri*), *Par Domain Protein 1* (*Pdp1ϵ*) and *clockwork orange* (*cwo*) by binding to their promoters [60, 61]. Conversely, the induced TIM and PER inhibit the activity of CLK-CYC in the nucleus, which allows the clock to be oscillated. The clock oscillation is also modulated by *Clk* transcription, which is first repressed by VRI and then activated by PDP1ϵ. CWO also directly activates transcription of *per*, *tim*, *vri*, *Pdp1ϵ* by binding to their promoters.

The timing of developmental transitions, such as molting and eclosion, are regulated by a circadian clock in some insects, in which the circadian clock appears to control ecdysteroid biosynthesis in the PGs. For example, in the blood-sucking bug *Rhodnius prolixus* and the leafworm *Spodoptera littoralis*, ecdysteroid titers fluctuate with a daily rhythm and such temporal changes control the timing of molting during development [62, 63]. In *Drosophila*, the timing of transition from pupae to adults is gated by the timing of ecdysteroid biosynthesis, which is under control of the circadian clock components in not only PG cells, but also in neuronal cells of the brain [64, 65]. By contrast, the relationship between ecdysteroids and circadian clocks has been largely unknown until recently, but some pioneer studies focusing on this issue have been reported in recent years.

**Fig. 3:**
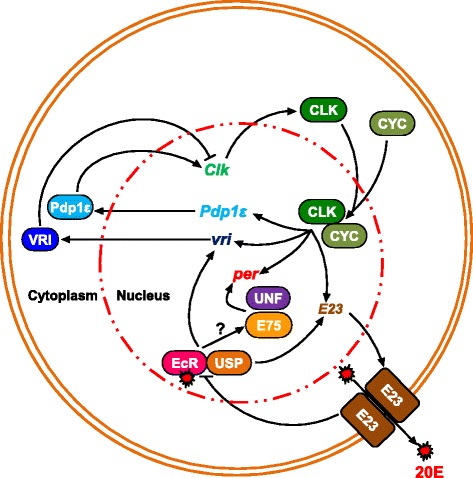
Scheme illustrating ecdysone signaling factors in the molecular machinery of the *Drosophila* circadian clock. The figure is modified from Itoh and Matsumoto [92]. The signal of 20-hydroxyecdysone (20E), the most biologically active ecdysteroid, is transduced primarily through the action of the specific receptor for 20E. This receptor is a heterodimer of Ecdysone receptor (EcR) and Ultraspiracle (Usp), which binds a specific DNA element when 20E is present. The 20E-bound form of EcR/Usp complex activates transcriptions of *vrille* (*vri*) and *Early gene at 23* (*E23*). The CLK-CYC also activates transcriptions of *period* (*per*), *vri* and *E23*. The E23 protein specifically negates the 20E response. Furthermore, this EcR-Usp complex starts the ecdysteroid cascade with the expression of *E75*. The E75 and UNF activate transcriptions of *per*

For example, *E75* and *unfulfilled* (*unf*; *DHR51*), which encode nuclear receptors, have been identified as components of the molecular clocks in the *Drosophila* pacemaker neurons, as knockdown of *E75* and *unf* in the clock neurons lengthen the free-running period [66]. E75 and UNF bind to *per* regulatory sequences and act together to enhance the CLK/CYC-mediated transcription of the *per* gene (Fig. 3) [66]. Notably, *E75* has also been recognized as a component of molecular clocks in other animals. For example, in the firebrat *Thermobia domestica*, a primitive insect, normal rhythmic expression of *E75* and *nuclear hormone receptor 3* (*HR3*) is required for the persistence of locomotor rhythms [67]. Interestingly, *HR3* and *E75* are orthologs of mammalian clock genes, *Rorα* and *Rev-erbα*. Despite these mechanistic divergences, the notion that *Rorα* and *Rev-erbα* homologs are integral to the molecular oscillators in both insects and mammals highlights the significance of transcriptional regulations via nuclear receptors in metazoan circadian clocks [66, 67].

In addition to nuclear receptor-mediated regulation, another type of the feedback loop of ecdysteroid signaling has been implicated in the *Drosophila* circadian clock by studies on a gene called *Early gene at 23* (*E23*) encoding the ABC transporter (Fig. 3) [68, 69]. The *E23* knockdown flies lengthen circadian period with an increased expression of the clock gene *vri. E23* and *vri* are positively regulated by 20E in pacemaker neurons, whereas E23 negatively regulates 20E-dependent signaling [69]. Considering that E23 protein depresses the 20E response in cultured cells [69], this ABC transporter might cause the reduction in intracellular level of 20E. Taken together, E23 forms its own feedback loop in the ecdysteroid response through the E23 function itself and ecdysteroid-mediated *vri* expression (Fig. 3) [69].

Consistent with the fact that 20E is involved in the regulation of the circadian clock, *EcR* is expressed in circadian neurons [70], and the double knockdown flies of *EcR* and *usp* exhibit the abnormal circadian phenotype [69]. It is therefore important to identify transcriptional targets of EcR/USP. *E75* and *E23* are the EcR-USP targets in the clock neurons [71]. A recent study has also reported that the microRNA *let-7* is a target of EcR/USP [72]. *let-7* is the evolutionarily-well conserved microRNA and involved in temporal regulation of development and physiology in many animals [73]. Importantly, *let-7* targets the crucial clock component CWO. The ecdysteroid-induced *let-7* regulates the circadian rhythm via repression of CWO, as up-regulation of *cwo* rescues the circadian clock phenotype in flies overexpressing the *let-7*-complex [72]. Taken together, ecdysteroid signaling has multiple functions in controlling the circadian clock in *Drosophila* adults at several levels of regulation, such as the transporter-mediated, transcriptional and post-transcriptional levels.

## Conclusions

There is a growing body of evidence of the importance of ecdysteroids in adult insects. Steroid hormones are small and fat-soluble bioactive molecules that can be easily circulated throughout the body and pass through the cell membrane into cells [5]. Steroid hormones, therefore, have the potential to rapidly and systemically orchestrate many types of cells in the whole body. It is feasible that ecdysteroid signaling is used to orchestrate individual biological events not only in developing animals but also in adults, although an actual benefit of signaling for adult insects has not been fully elucidated. Curiously, steroid hormones are involved in controlling germline development [74] and circadian rhythms in mammals [75], implying that the functions of steroid hormones in adults are, at least in part, evolutionarily conserved.

One important unanswered question is the ecdysteroidogenic cell(s) or organ(s) (other than the ovary) responsible for biosynthesizing ecdysteroids after eclosion. While the PG is the organ responsible for biosynthesizing ecdysteroids during larval and early pupal stages, the PG degenerates during pupal development and is eventually lost in the adult stage [76–79]. It is possible that the ovary is the source of circulating ecdysteroids in adult female hemolymph, as has been shown in the cockroach *Blattella germanica* [77]. Although the ovariectomized *Blattella* female exhibits a reduced ecdysteroid titer, a substantial amount of the hemolymph ecdysteroids remain [77]. In the case of male adults, while several recent studies have identified the accessory gland as a site of ecdysteroid production [80, 81], it is unclear whether accessory gland-producing ecdysteroids systemically act in the whole body. Neuronal subpopulations are a strong candidate for the unidentified adult ecdysteroidogenic cells. 20E is detected in the brain of *Drosophila*, and its expression is regulated by the *clock* gene [72]. Second, some ecdysteroidogenic enzymes are expressed in the brain in the honeybee *Apis mellifera* [82] and in *Drosophila* (Yuko Shimada-Niwa, Sora Enya and R.N., unpublished observation). Third, a clock neuron-specific knockdown of the ecdysteroidogenic gene *phantom* exhibits an abnormal free-running period in *Drosophila* [69]. It should be noted that vertebrate nervous systems can biosynthesize *de novo* steroids, known as neurosteroids, which modulate neuronal activities [83, 84]. By extension, the possibility that *de novo* biosynthesized ecdysteroids also act as neuromodulators and are required for adult neuronal functions represents an attractive hypothesis. To understand the regulatory mechanisms controlling production of ecdysteroids in adult flies, it is important to examine where ecdysteroidogenic enzyme genes are expressed, and how their expression and activity are regulated at cellular resolution.

Another interesting issue to be addressed is whether ecdysteroids regulate GSCs and the circadian clock cooperatively with juvenile hormone (JH), which is also a key insect hormone that regulates molting and metamorphosis [85, 86]. It is well known that JH plays a crucial role in controlling adult ovarian maturation in many insects. In female *Drosophila* there is a functional interaction between 20E and JH to regulate ovarian maturation and oviposition [87]. A role of JH in regulating the circadian clock has also been implied by a study on the gene *takeout*, which encodes the JH binding protein. *takeout* is essential for a circadian output pathway that conveys temporal information to feeding-relevant metabolism and activities [88]. However, whether and how GSCs and circadian clocks are regulated by a crosstalk between 20E and JH is still an intriguing open question.

In addition to the role of ecdysteroids in the adult stage summarized in this paper, other ecdysteroid-dependent biological events in the adult stage have also been reported, such as stress resistance [54], lifespan [14, 89, 90], and innate immunity [91]. A number of studies on vertebrates have revealed that the actions of steroid hormones play crucial roles in adult homeostasis. In this sense, further investigation of the roles of ecdysteroids in adult insects is needed to establish a secure foundation for the use of insects as model organisms in steroid hormone research. Considering the recent remarkable advances in knowledge and resources of ecdysteroid biosynthesis and signaling, it is likely that additional essential adult events that are regulated by ecdysteroids will be found in the future.

## Abbreviations

20E: 20-hydroxyecdysone
*Br*: *Broad*
*Clk*: *Clock*
*cwo*: *clockwork orange*
*cyc*: *cycle*
CNS: Central nervous system
CyC: Cyst stem cell
*E23*: *Early gene at 23*
*E75*: *Ecdysone-induced protein 75*
*E78*: *Ecdysone-induced protein 78*
*EcR*: *Ecdysone receptor*
GSC: Germline stem cell
*HR3*: *Nuclear hormone receptor 3*
ISWI: An intrinsic epigenetic factor
JH: Juvenile hormone
*Pdp1ϵ*: *Par Domain Protein 1*
*per*: *period*
PG: Prothoracic gland
PGC: Primordial germ cell
*Rorα*: *RAR-related orphan receptor alpha*
*SREBP*: *Sterol regulatory element-binding proteins*
*tim*: *timeless*
*unf*: *unfulfilled*
*vri*: *vrille*
